# Inheritance of STING mosaicism in two half-siblings

**DOI:** 10.1007/s10875-024-01768-9

**Published:** 2024-07-30

**Authors:** Alix de Becdelièvre, Laurye-Anne Eveillard, Beata Wolska-Kuśnierz, Marie-Louise Frémond, SAVI study group, Laureline Berteloot, Yanick J Crow, Clémence David, Alice Hadchouel, Bénédicte Neven, Pierre Quartier, Gillian I Rice, Luis Seabra, Anne Welfringer-Morin

**Affiliations:** 7 Pediatric Radiology Department, Hôpital Necker-Enfants Malades, AP-HP.Centre Université de Paris, Paris, France; 8 MRC Human Genetics Unit, Institute of Genetics and Cancer, University of Edinburgh, Edinburgh, United Kingdom; 9 Pediatric Pulmonology Department, Hôpital Necker-Enfants Malades, AP-HP.Centre Université de Paris, Paris, France; 10 INEM, INSERM U1151, Paris, France; 11 Laboratory of Immunogenetics of Pediatric Autoimmunity, Imagine Institute, INSERM UMR 1163, Paris, France; 12 Division of Evolution and Genomic Sciences, School of Biological Sciences, Faculty of Biology, Medicine and Health, University of Manchester, Manchester Academic Health Science Centre, Manchester, M13 9PT, United Kingdom; 13 Dermatology Department, Hôpital Necker-Enfants Malades, AP-HP.Centre Université de Paris, Paris, France; 1 AP-HP, Laboratoire de Génétique, Hôpital Henri Mondor, Créteil, France; 2 University Paris Est Créteil, INSERM IMRB Créteil, France; 3 Paediatric Hematology-Immunology and Rheumatology Unit, Necker-Enfants Malades Hospital, AP-HP, Paris, France; 4 Immunology Department, Children’s Memorial Health Institute, Warsaw, Poland; 5 Université Paris Cité, Paris, France; 6 Laboratory of Neurogenetics and Neuroinflammation, Imagine Institute, INSERM UMR1163, Paris, France

## To the Editor

STING-associated vasculopathy with onset in infancy (SAVI) was described ten years ago as a severe autosomal dominant autoinflammatory disorder associated with constitutive type I interferon (IFN) signalling^1^. SAVI is caused by gain-of-function mutations in *STING1* (previously known as *TMEM173*), which encodes STING (stimulator of IFN genes), a core adaptor protein of the cytoplasmic DNA signalling pathway that, upon activation, leads to the induction of type I IFN and NF-κB signalling. Causative heterozygous pathogenic variants can arise *de novo*^1^ or be inherited. Recently, autosomal recessive disease due to homozygosity for a single amino acid substitution (c.841C>T p.(Arg281Trp)) has been described^2^.

Advances in DNA sequencing have expanded the molecular understanding of genetic autoinflammatory diseases, including by highlighting the possibility of somatic mosaicism, even in severe and / or neonatal onset of such disorders^3,4^. In the context of SAVI, somatic mosaicism was suggested in one of the first patients described who had higher mutation rate in keratinocytes than in leukocytes^1^. Here, we report a family with documented maternal *STING1* mosaicism and transmission of the pathogenic variant to two maternal half-siblings presenting with typical features of SAVI.

**Figure 1:**
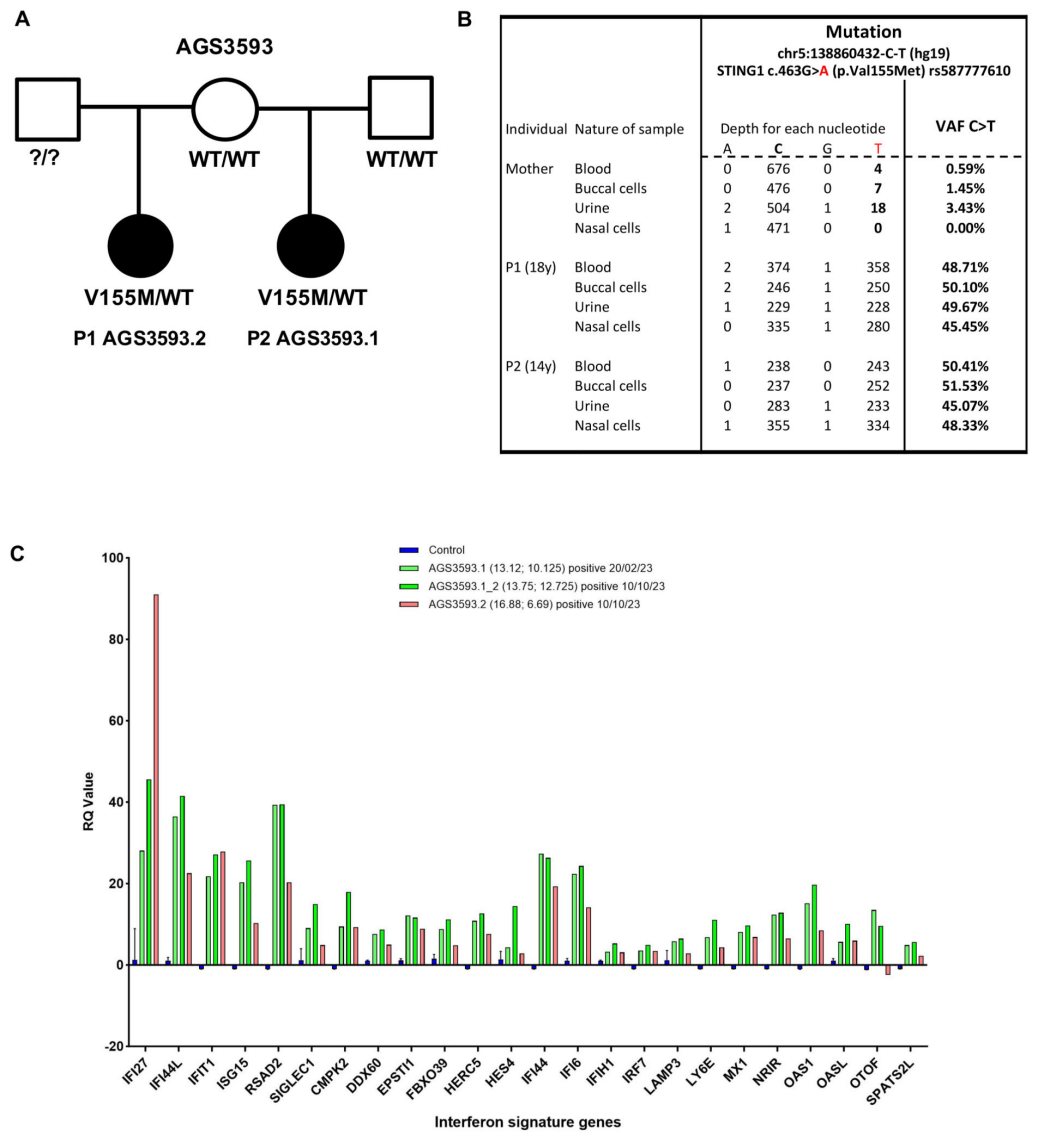
Inherited mosaicism for the *STING1* V155M mutation in two-half siblings (A) Pedigree. Circles (females) and squares (males) blackened and clear indicate respectively symptomatic and asymptomatic individuals. The status for the V155M mutation in *STING1* in the whole blood is specified for each individual. WT: wild-type. (B) Assessment of *STING1* mutation c.463G>A (p.Val155Met) by next-generation sequencing (NGS) in the mother and the probands in different tissues. (C) Interferon-stimulated gene (ISG) expression measured in the whole blood from controls and the two patients (AGS3593.1 and AGS3593.2) using a 24 ISG panel on a NanoString platform to calculate the interferon score.

P1 and P2 were born to non-consanguineous parents, sharing the same mother but with different fathers (Figure 1A). P1 (AGS3593.2) was diagnosed with polyarthritis at the age of 6 years. Three years later she became dyspnoeic on exertion, and chest computed tomography (CT) scan showed fibrosing interstitial lung disease (ILD). She had experienced only very few episodes of recurrent fevers, and no skin vasculopathy. P2 (AGS35393.1) presented with early-onset polyarthritis of the small joints and severe chilblain like lesions of the legs. Her chest CT scan showed symmetric ILD with radiologically defined features of progression to fibrosis. P1 and P2 had received several lines of immunosuppressants, including high dose steroids, with no clear efficacy.

Genetic analysis using a next-generation sequencing (NGS) panel identified a known pathogenic heterozygous mutation in the *STING1* gene (NM_198282.4): c.463G>A, p.(Val155Met: V155M), in P1, and subsequently in P2. Familial segregation was performed and the mutation was not found in the whole blood of the mother or father of P2. Due to the finding of the same variant in P1 and P2, we considered the possibility of somatic mosaicism in the mother, and performed a second genetic analysis using an NGS targeted panel on DNA samples from both patients and their mother in different tissues (i.e. blood, urine, buccal swab, nasal swab). The threshold in variant allele frequency (VAF) which is usually at 20% to detect constitutional variants, was lowered to 0.1% to allow detection of a low percentage of the mutant. Bam files were visualized with Integrative Genomics Viewer (IGV) (Figure S1B). Using two benign single nucleotide polymorphisms (SNPs) in the *STING* gene as controls, we evaluated the technical background noise as <0.5% of the reads in the mother’s samples in this region.. In doing so, we detected the p.(Val155Met) mutation in the heterozygous status in all tissues assessed from P1 and P2 (Figure 1B). The variant was not detected in the mother’s nasal cells. The signal was not distinguishable from technical background in her blood cells. Interestingly, the p.(Val155Met) mutation was detected at 3.43% in a urine sample from the mother, while detected at lower percentages in the buccal cells..

Taken together, our data show that the two half-sisters with typical SAVI inherited a constitutive *STING1* mutation from their asymptomatic mother who demonstrated low level somatic mosaicism for the same variant.

Following the diagnosis of SAVI, P1 and P2 were started on a JAK inhibitor at the age of 14 and 12 years respectively. At last contact, 2 years after initiation of JAK inhibition, a mild improvement was observed in the severe skin vasculopathy of P2. The lung status was stabilised in both P1 and P2, and their joint inflammation was in remission. IFN scores in whole blood remained high despite JAK inhibition (Figure 1C), as previously observed in other patients with SAVI.

Here we report genetic inheritance of SAVI in two half-siblings with SAVI explained by maternal somatic mosaicism. This observation highlights the complexity of the genetic landscape of Mendelian autoinflammatory diseases, and supports the use of low VAF threshold next-generation sequencing in genetically unresolved patients presenting with the typical phenotype of a known monogenic autoinflammatory disorder, and the need to counsel the possibility of recurrence even where a mutation in STING1 has apparently arisen *de novo*.

## Supplementary Material

Supplementary

Supplementary figure 1
