# Supplementary material for: Inheritance of STING mosaicism in two half-siblings
Source: J Clin Immunol. Author manuscript; Available in PMC 2024 Aug 13. (PMC7616363; doi:10.1007/s10875-024-01768-9)
Supplement: Supplementary [file EMS197976-supplement-Supplementary.docx]

**Title: Inheritance of STING mosaicism in two half-siblings**

**Authors:**

Alix de Becdelièvre^1,2^, Laurye-Anne Eveillard^3^, Beata Wolska-Kuśnierz^4^, Marie-Louise Frémond^3,5,6#^ and SAVI study group

SAVI study group: Laureline Berteloot^7^, Yanick J Crow^5,6,8^, Clémence David^6^, Alice Hadchouel^9,10^, Bénédicte Neven^3,5,11^, Pierre Quartier^,5,11^, Gillian I Rice^12^, Luis Seabra^6^, Anne Welfringer-Morin^13^

^#^Corresponding author

**Affiliations:**

^1^AP-HP, Laboratoire de Génétique, Hôpital Henri Mondor, Créteil, France

^2^University Paris Est Créteil, INSERM IMRB Créteil, France

^3^Paediatric Hematology-Immunology and Rheumatology Unit, Necker-Enfants Malades Hospital, AP-HP, Paris, France

^4^Immunology Department, Children’s Memorial Health Institute, Warsaw, Poland

^5^Université Paris Cité, Paris, France

^6^Laboratory of Neurogenetics and Neuroinflammation, Imagine Institute, INSERM UMR1163, Paris, France

**Contributor Information**

For the SAVI group:

^7^Pediatric Radiology Department, Hôpital Necker-Enfants Malades, AP-HP.Centre Université de Paris, Paris, France

^8^MRC Human Genetics Unit, Institute of Genetics and Cancer, University of Edinburgh, Edinburgh, United Kingdom

^9^Pediatric Pulmonology Department, Hôpital Necker-Enfants Malades, AP-HP.Centre Université de Paris, Paris, France

^10^INEM, INSERM U1151, Paris, France

^11^Laboratory of Immunogenetics of Pediatric Autoimmunity, Imagine Institute, INSERM UMR 1163, Paris, France

^12^Division of Evolution and Genomic Sciences, School of Biological Sciences, Faculty of Biology, Medicine and Health, University of Manchester, Manchester Academic Health Science Centre, Manchester, M13 9PT, United Kingdom

^13^Dermatology Department, Hôpital Necker-Enfants Malades, AP-HP.Centre Université de Paris, Paris, France

**Supplementary material**

**Supplementary Figure 1. Characterisation of STING V155 mutation mosaicism**

1. Alignment of bam files generated from DNA extracted from urine using the Integrative Genomics Viewer (IGV) software. The genomic reference sequence is reversed compared to the gene's HGVS nomenclature due to the orientation of *STING1* on the chromosome.
2. Observation of read depths on the mutation site and two polymorphisms employed as controls. The read depth is provided for each alternative nucleotide at the specified position. For the two benign polymorphisms used as controls to mitigate read errors and DNA contamination, the background noise was evaluated by relating the depth of the most represented inaccurate base (in bold) to the total depth on the position. The background noise for each possible alternative nucleotide does not surpass 0.55% of reads. The VAF (variation allele frequency) column denotes the proportion of mutations in total reads, with a rate nearing 50% indicating heterozygotes. However, in the mother's blood, whether the VAF of 0.59% represents noise or true mosaic is not discernable. Conversely, in buccal cells and urine, mosaic levels exceed the background.
