# Supplementary material for: Inheritance of STING mosaicism in two half-siblings
Source: J Clin Immunol. Author manuscript; Available in PMC 2024 Aug 13. (PMC7616363; doi:10.1007/s10875-024-01768-9)
Supplement: Supplementary figure 1 [file EMS197976-supplement-Supplementary_figure_1.pdf]

Supplemental Figure 1. Characterisation of STING V155 mutation mosaicism

A

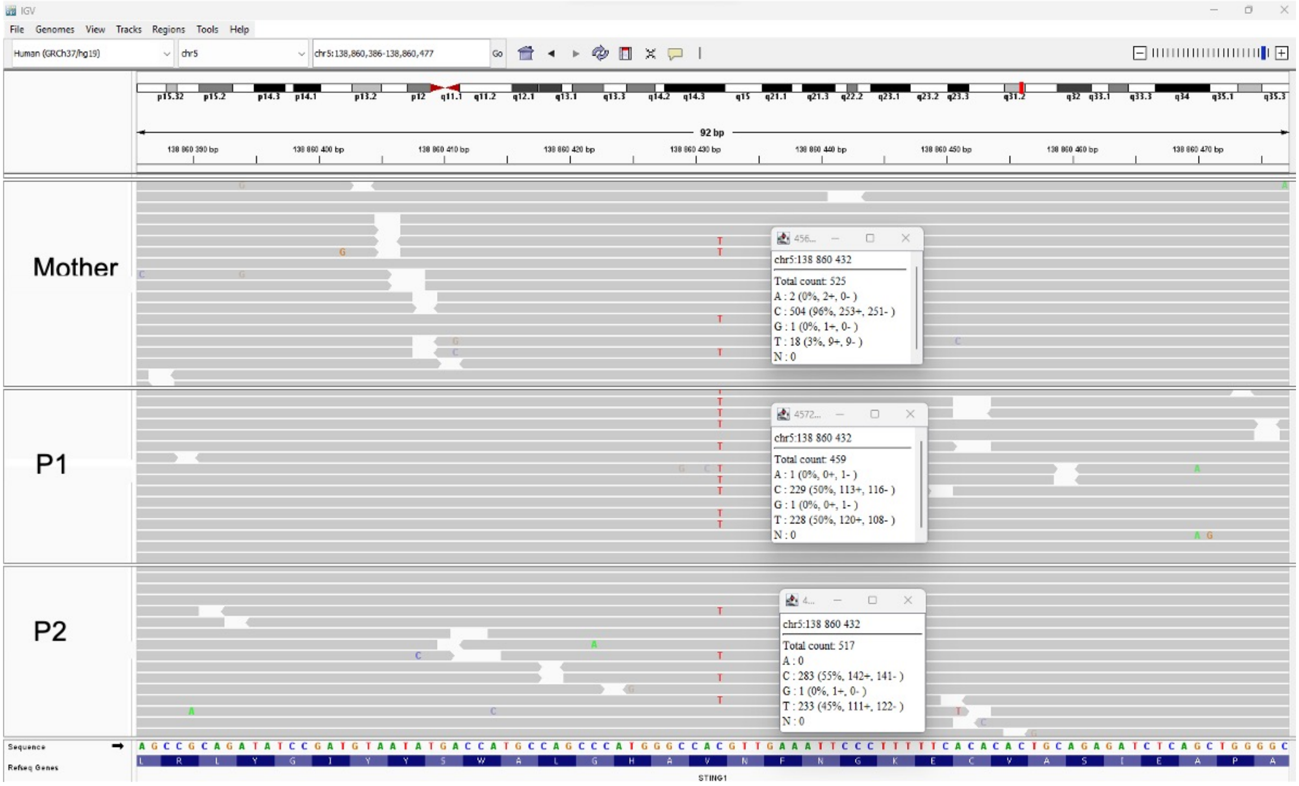

B

Individual	Nature of sample	Controls SNPs<br>chr21:47703649-G-A (hg19)<br>MCM3AP: c.1323C>T (p.Asn441=) rs1240925<br>Depth for each nucleotide<br>A	C	G	T	Max. noise	chr5:138861146-C-G (hg19)<br>STING1: c.144G>C (p.Val48=) rs7447927<br>Depth for each nucleotide<br>A	C	G	T	Max. noise	Mutation<br>chr5:138860432-C-T (hg19)<br>STING1 c.463G>A (p.Val155Met)<br>rs587777610<br>Depth for each nucleotide<br>A	C	G	T	VAF C>T
Mother	Blood	1	0	867	4	0.46%	0	643	0	0	0%	0	676	0	**4**	**0.59%**
	Buccal cells	0	0	648	**3**	0.46%	0	465	**1**	0	0.15%	0	476	0	**7**	**1.45%**
	Urine	0	0	695	**1**	0.14%	0	451	**1**	0	0.15%	2	504	1	**18**	**3.43%**
	Nasal cells	0	0	664	**1**	0.15%	1	471	**1**	0	0.15%	1	471	0	**0**	**0%**
P1 (18y)	Blood	472	0	498	0	0%	0	359	390	0	0%	2	374	1	358	**48.71%**
	Buccal cells	375	0	305	0	0%	0	254	256	**1**	0.15%	2	246	1	250	**50.10%**
	Urine	258	1	280	**3**	0.55%	0	161	228	0	0%	1	229	1	228	**49.67%**
	Nasal cells	335	0	302	0	0%	0	331	274	0	0%	0	335	1	280	**45.45%**
P2 (14y)	Blood	1	0	586	**1**	0.17%	0	244	259	0	0.15%	1	238	0	243	**50.41%**
	Buccal cells	0	0	596	0	0%	0	283	261	0	0%	0	237	0	252	**51.53%**
	Urine	0	0	638	**1**	0.15%	0	270	219	0	0%	0	283	1	233	**45.07%**
	Nasal cells	0	0	921	**2**	0.22%	**1**	328	333	0	0.15%	1	355	1	334	**48.33%**
